# A rare case of ectopic adrenal gland in an adult inguinal cord lipoma: Case report and literature review

**DOI:** 10.1016/j.ijscr.2024.109390

**Published:** 2024-02-15

**Authors:** Souad Ghattas, Hani Maalouf, Hind Rahban, Gihan Nakhleh, Ziad El Rassi, Antoine El Asmar

**Affiliations:** a Department of General Surgery, Saint Georges Hospital University Medical Center, Beirut, Lebanon; b Head of Laboratory Department, Lebanese American University Medical Center, Beirut, Lebanon; c Department of Anesthesiology, Saint Georges Hospital University Medical Center, Beirut, Lebanon; d Head of General Surgery Department, Saint Georges Hospital University Medical Center, Beirut, Lebanon

**Keywords:** Ectopic adrenal, Inguinal hernia repair, Cord lipoma, Case report

## Abstract

**Introduction:**

Ectopic adrenocortical tissue is defined as the presence of accessory adrenal cortex tissue outside the suprarenal location of the adrenal glands. It is not an infrequent finding during inguinal operations in infants, however, its incidence in adults is found to be less than 1 %.

**Case:**

We report a case of ectopic adrenal tissue incidentally found in a cord lipoma of a 68-year-old man, presenting for elective inguinal hernia repair.

**Clinical discussion:**

In the literature, the majority of cases of ectopic adrenocortical tissue are reported during groin surgeries in children. After the first few years of life, it normally regresses, but in a few uncommon cases like ours, it might continue long into adulthood. The condition can have several theoretical clinical implications that need to be considered by surgeons. Adrenal insufficiency can occur if the ectopic adrenal tissue is the only adrenal tissue in the patient along with a potential for neoplastic transformation in cases of persistence of ectopia.

**Conclusion:**

However, studies have shown no evidence of endocrine or oncologic complications after excision or persistence of the ectopic adrenal gland. Consequently, no investigations or treatments are indicated.

## Highlights

• Adrenal ectopy is a rare histopathologic diagnosis that can be made after inguinal hernia repair in adults.
• The condition can have several theoretical clinical implications that need to be considered by surgeons. Adrenal insufficiency can occur if the ectopic adrenal tissue was the only adrenal tissue in the patient along with a potential for neoplastic transformation in cases of persistence of ectopia.
• There is no evidence of endocrine or oncologic complications after excision. Consequently, no specific investigations or treatments measures are indicated postoperatively in these patients.

## 1 Introduction

The adrenal glands are retroperitoneal organs located above the kidneys. During embryogenesis, abnormal migration of mesoderm or ectoderm can alter adrenal and testicular development, resulting in abnormal localization of adrenal tissue, a rare condition known as adrenal heterotopia [1]. Ectopic adrenocortical tissue is usually discovered during groin surgery in children and disappears after a few years of life [2]. Therefore, the incidence of ectopic adrenal tissue in adults is less than 1 % [1]. The work was reported according to his SCARE standards [3].

Here we report a rare case of ectopic adrenocortical tissue in an inguinal cord lipoma in an adult patient who underwent hernia repair.

## 2 Case report

A 68-year-old male patient presented for elective bilateral inguinal hernias repair.

He denied any symptoms of bowel obstruction or endocrine dysfunction. Past medical history was significant for hypertension and transient ischemic attack. His past surgical history included double J catheter placement 10 years ago followed by lithotripsy for nephrolithiasis and submucosal resection of the nose. Family history was non-significant. Physical examination showed bilateral reducible inguinal hernias.

He was admitted for elective bilateral open inguinal hernia repair with mesh. Surgery was performed under spinal anesthesia. During dissection, bilateral large lipomas of the cord were identified, highly ligated, excised, and sent to pathology. Bilateral direct inguinal hernias were seen, reduced with a purse-string suture and oversewing of the overlying muscular tissue, and Lichtenstein herniorrhaphy was performed. The patient was discharged home on day one post-surgery after an uneventful hospital stay.

**Fig. 1:**
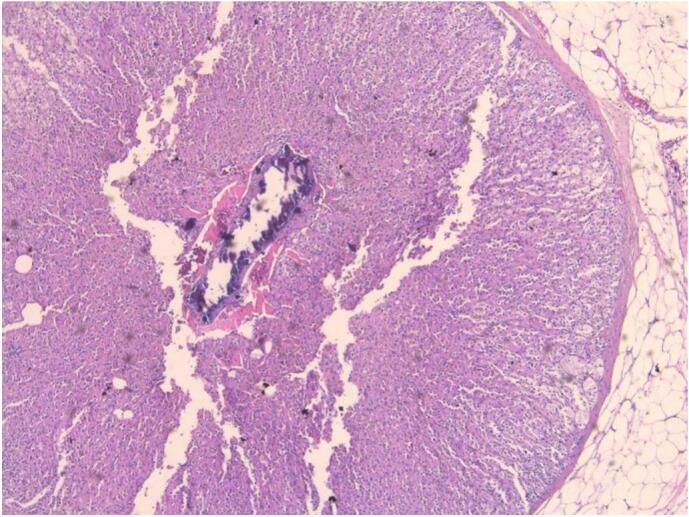
Adrenocortical tissue found in hernia lipoma, low power view.

One week later, the histopathology report came back reporting the presence of ectopic adrenal tissue in the right cord lipoma. A gross examination of the lipoma revealed a membrane fatty tissue fragment measuring 1.5 × 1.5 × 0.3 cm displaying a focally orange nodule measuring 0.3 × 0.3 cm (Fig. 1).

## 3 Discussion

Ectopic adrenal tissue is the presence of adrenal tissue outside of the adrenal glands [4]. It was first described by Morgagni in 1740 from the spermatic cord of a child [1]. It occurs when pieces of adrenal gland tissue slough off during development [1]. Adrenocortical tissue develops from the mesoderm during the fourth and fifth weeks of pregnancy and is found anywhere along the descent of gonadal tissue, but it can stop at any point along this pathway [5]. Ectopic adrenal tissue has been identified in several locations around the celiac axis (32 %), in the broad ligament (23 %), in the testicular appendage (7.5 %), and most rarely in the spermatic cord (1–9.3 %) was also recognized [4].

No case of ectopic adrenal cortex with medullary tissue has been recorded in the literature. This is explained by the different embryological origins of the adrenal cortex and medulla oblongata. The cerebral component originates from the mesoderm located between the mesenteric roots and the developing gonads, whereas the medulla originates from the neural crest ectoderm, invades the developing cerebral cortex, and eventually forms a final site later in development. Because the gonads and cortex are in close physical proximity, cortical tissue may move mechanically during the descent of the developing gonads [2].

In our patient, ectopic adrenocortical tissue was found in a spermatic cord lipoma. Lipomas are a common finding during hernia repair. These benign fat masses are considered clinically important because they cause hernia-like symptoms in the absence of a true hernia [4].

The literature reports the majority of cases in which ectopic adrenocortical tissue occurred during groin surgery in children. This condition usually resolves within the first few years of life, but in rare cases like ours, it can persist into adulthood [5]. The actual incidence of inguinal adrenal dystopia may be underestimated because it resembles the surrounding adipose tissue. The diagnosis is then made based on histopathology [4].

The presence of ectopic adrenocortical tissue in the groin may have several clinical implications [4]. Theoretically, adrenal insufficiency could occur if a patient had only ectopic adrenal tissue or if the adrenal tissue removed was hyperactive and inhibited normal adrenal gland function. In addition, with persistent ectopia in the groin, there is a possibility of neoplastic changes in the tissue [6]. Of note, no cases of neoplastic changes in patients with persistent ectopia have been reported, and no cases of adrenal insufficiency after removal of ectopic adrenocortical tissue have been reported in the literature [5]. As a result, adrenal ectopia has little clinical significance and does not require further investigation or treatment after resection [5]. Based on these theoretical consequences, surgeons are often directed to excise lesions suggestive of ectopic adrenocortical tissue during groin surgery [2]. However, routine aggressive search for ectopic gonads during inguinal procedures is not necessary because cutting the spermatic cord carries the risk of vascular injury [2].

## 4 Conclusion

Ectopic adrenocortical tissue is an exceedingly rare histopathological diagnosis after inguinal hernia repair in adults, particularly within the inguinal cord. Its identification is incidental, facilitated by the submission of surgical specimens for histopathological evaluation. Consequently, its incidence is underestimated. This condition may have several theoretical clinical implications that the surgeon must consider. However, so far there is no evidence of endocrine or tumor-based complications after resection. Therefore, no special postoperative testing or treatment measures are indicated for these patients.

## Informed consent

Written informed consent was obtained from the patient for publication and any accompanying images. A copy of the written consent is available for review by the Editor-in-Chief of this journal on request.

## Ethical approval

Case report approved for publishing by ethical committee at Saint George hospital University Medical Center, and Head of General Surgery division, Beirut, Lebanon, 2023.

## Funding

Faculty of Medicine and Medical Sciences, University of Balamand, Lebanon.

## Author contribution

Souad Ghattas MD (First Author), Hani Maalouf, Gihan Nakhleh, Hind Rahban, Ziad El Rassi, Antoine Asmar MD (Corresponding Author).

## Guarantor

Souad Ghattas.

## Provenance and peer review

Not commissioned, externally peer-reviewed. This work has been reported in line with the SCARE criteria: Sohrabi C, Mathew G, Maria N, Kerwan A, Franchi T, Agha RA. The SCARE 2023 guideline: updating consensus Surgical Case Report (SCARE) guidelines. Int J Surg Lond Engl. 2023;109(5):1136.

## Conflict of interest statement

The authors report no conflicts of interest.
